# Advantages of intraoral and transconjunctival approaches for posterior displacement of a fractured zygomaticomaxillary complex

**DOI:** 10.1186/s40902-016-0085-x

**Published:** 2016-10-25

**Authors:** Ji Yong Yoo, Jang Won Lee, Seung Jae Paek, Won Jong Park, Eun Joo Choi, Kyung-Hwan Kwon, Moon-Gi Choi

**Affiliations:** 1 Department of Oral and Maxillofacial Surgery, College of Dentistry, and Dental Hospital, Wonkwang University, 460, Iksandae-ro, Iksan, Jeollabuk-do South Korea; 2 Department of Oral and Maxillofacial Surgery, College of Dentistry, Wonkwang Dental Research Institute, Wonkwang University, Iksan, South Korea

**Keywords:** Displacement direction, Surgical approach, Zygomaticomaxillary complex (ZMC) fracture

## Abstract

**Background:**

Fracture of the zygomaticomaxillary complex (ZMC) is one of the most common facial injuries. A previous study has performed 3D analyses of the parallel and rotational displacements that occur in a fractured ZMC. However, few studies have investigated adequate fixation methods according to these displacements. Here, we assessed whether specific approaches and fixation methods for displacement of ZMC fractures produce esthetic results.

**Methods:**

Hospital records and pre- and post-surgical computed tomographic scans of patients treated for ZMC fractures at the Department of Oral and Maxillofacial Surgery, College of Dentistry, Wonkwang University, between January 2010 and December 2015, were selected. Data were analyzed according to the direction of displacement and post-reduction prognosis using a 3D software.

**Results:**

With ZMC fractures, displacement in the posterior direction occurred most frequently, while displacement in the superior-inferior direction was rare. A reduction using a transconjunctival approach and an intraoral approach was statistically better than that using an intraoral approach, Gillies approach, and lateral canthotomy approach for a posterior displacement (*P* < 0.05).

**Conclusions:**

When posterior displacement of a fractured ZMC occurs, use of an intraoral approach and transconjunctival approach simultaneously is recommended for reducing and fixing the displaced fragment accurately.

## Background

Fracture of the zygomaticomaxillary complex (ZMC) is one of the most common facial injuries [1, 2]. Treatment of ZMC injury has improved due to various reduction methods and the development of miniplates and screws. Treatment of ZMC fractures consists of reduction and fixation of the dislocated bone fragments to their original location. Most ZMC bones are successfully repositioned by reduction using an intraoral approach, transconjunctival approach, and Gillies approach [1].

Toriumi et al. have described complication of the ZMC injury, such as asymmetry of the zygoma, trismus, diplopia, limitation of eye movement, and hypoesthesia [3]. Reduction accuracy of fractured bone fragments has been the major focus of most studies on facial asymmetry that resulted from zygoma asymmetry [4]. Ellis et al. have reported that facial asymmetry that did not exceed 2 mm was difficult to perceive, even by experienced clinicians, and that most asymmetries after reduction of ZMC were acceptable [5]. When bone fragments were precisely reduced, no asymmetry was reported [1].

Although numerous studies on surgical approaches and fixation methods for ZMC fractures have been published, few studies have analyzed the degree of ZMC displacement quantitatively [6–10]. Toriumi et al. reported that displacement around the superior-inferior axis was the most frequent and that displacement around the posterior-anterior axis was rare in ZMC fractures [3]. However, there have been no studies determining the appropriate surgical approach according to these directions. Therefore, here, we set out to re-classify ZMC fracture displacement according to the maximum projection coordinates of the zygoma, in order to determine which specific approach for treating displacement of ZMC fractures would result in less than a 2-mm deviation.

## Methods

### Subjects

A total of 102 adults were chosen to participate in this study. The patient group had reduction surgery after being admitted to the Department of Oral and Maxillofacial Surgery of Wonkwang University Dental Hospital or Medical Hospital due to ZMC fracture. Subjects in the patient group had received cone-beam computed tomography (CBCT) or multiple-detector computed tomography (MDCT) before and after the open reduction surgery. Inclusion criteria for the patient group were the absence of previous ZMC or maxilla fractures, diagnosis of a unilateral ZMC fracture, and open reduction surgery after the injury.

The intraoral approach, Gillies approach, transconjunctival approach, and lateral canthotomy approach (which we defined as all methods involving fixation of the frontozygomatic suture) were used for reduction. Bone fragments were fixated with titanium or biodegradable plates. Fixation locations were zygomaticomaxillary buttress, frontozygomatic buttress, and infraorbital rim; 1–3 of these points were fixed during reduction surgery.

### Methods

Hospital records and pre- and post-surgical computed tomographic scans of patients treated for ZMC fractures at the Department of Oral and Maxillofacial Surgery, College of Dentistry, Wonkwang University, between January 2010 and December 2015 were selected. Images were analyzed according to the direction of displacement, fixation methods used, and post-reduction prognosis, using a 3D software (OnDemand3D; Cybermed Inc., Seoul, Korea).

**Fig. 1:**
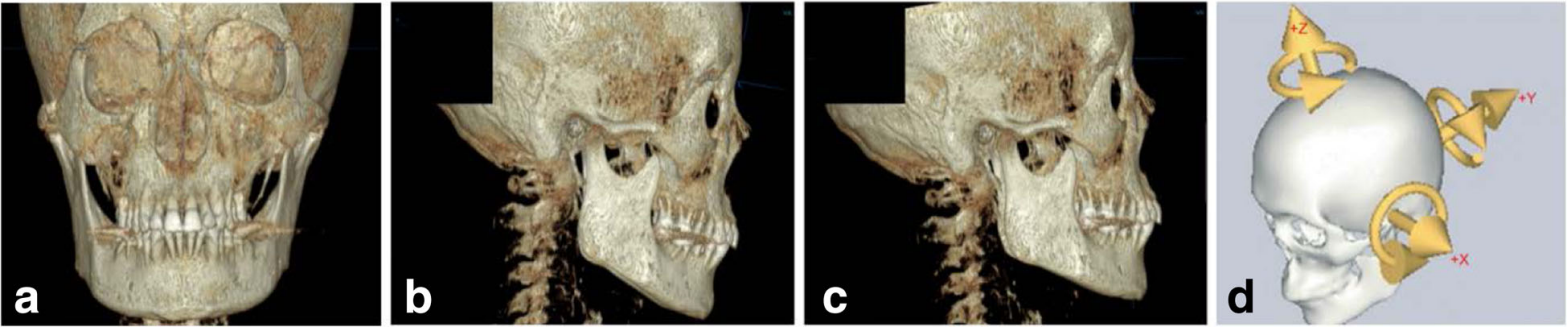
Re-orientation of the *x*, *y*, and *z* planes. Constructed coordinate system used. **a** The NFZ plane was composed of the right and left frontozygomatic points and nasion. The frontozygomatic points were defined as the most anterior point of the frontozygomatic suture. See the horizontal line via nasion. **b** Before re-orientation. **c** After re-orientation. **d** The *X*-axis (transverse axis) is a line parallel to the frontozygomatic (FZ) line. The *Y*-axis (antero-posterior axis) is a line perpendicular to the FZ line while parallel to the right Frankfort horizontal (R FH) plane. The *Z*-axis is perpendicular to both the FZ line and R FH plane

We used images obtained using the CBCT system at Wonkwang University Dental Hospital and the MDCT system at Wonkwang University Medical Hospital. We analyzed CT images in Digital Imaging and Communications in Medicine (DICOM) format using a 3D software. 3D coordinates were set up for each subject, and cephalometric landmarks were designated. The nasofrontozygomatic (NFZ) plane was used as a reference plane of the skull base. The NFZ plane was composed of the right and left frontozygomatic points and nasion. The frontozygomatic points were defined as the most anterior point of the frontozygomatic suture. The coordinate origin (0, 0, 0) was set on N. Based on the origin, coordinates were constructed in the *x*, *y*, and *z* planes (Fig. 1). The *X*-axis (transverse axis) was a line parallel to the frontozygomatic (FZ) line. The *Y*-axis (antero-posterior axis) was a line perpendicular to the FZ line and parallel to the right Frankfort horizontal (R FH) plane. The *Z*-axis was perpendicular to both the FZ line and R FH plane. Using this coordinate system, three planes were defined. The midsagittal plane was defined as a plane perpendicular to the R FH plane and NFZ line while passing through the origin. The horizontal plane (Frankfort plane) was defined as a plane passing through the right porion (R Po), right orbitale (R Or), and left orbitale (L Or). The coronal plane was defined as a plane perpendicular to the horizontal and midsagittal planes and passing through the origin.

**Fig. 2:**
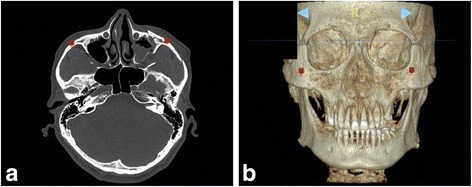
The zygoma landmark was marked at the most antero-lateral point. **a** In axial view. **b** In 3D view

To compare the amount of asymmetry between the ZMC fracture site and the opposite non-displaced site, the distance from the landmarks on the right and left zygomas to the midsagittal, horizontal, and coronal planes was measured. The zygoma landmark was marked at the most antero-lateral point in axial views (Fig. 2). The amount of asymmetry after ZMC surgery was measured using the same method. We acquired point values of the antero-posterior (AP), medio-lateral (ML), and superior-inferior (SI) directions.

Next, we analyzed these data according to the direction of displacement and post-reduction prognosis. The patient group was divided based on the amount of deviation (>2 mm or <2 mm). Ellis et al. have reported that facial asymmetry that did not exceed 2 mm was difficult to perceive, even by experienced clinicians, and that most asymmetries after reduction of ZMC were acceptable [5]. Therefore, a deviation of less than 2 mm was considered to be an acceptable standard for hard tissue reduction.

Preparation and measurement of all coordinates were repeated again twice by the same investigator, to prevent intra-observer error. Intra-observer error between the two measurements was verified using a paired *t* test. Crosstabulation using the chi-squared test was performed to assess the significance of differences between the measurements. Statistical analysis was conducted using SPSS software (SPSS version 21.0; SPSS Inc., Chicago, IL, USA) with a 95 % reliability.

## Results

### Intra-observer error test

Intra-observer error between the first pointing and the second pointing was verified using a paired *t* test (Table 1). The mean differences between the first and second pointing were very slight (less than 0.5 mm). Therefore, we concluded that there was no intra-observer error between these measurements, and the first pointing data were used in this study.

**Table 1:** Result of the paired *t* test between the first pointing and the second pointing

Measurement (mm)	The 1st pointing	The 2nd pointing
	Mean	Mean
Lt. AP	17.70	17.88
Lt. ML	54.52	54.62
Lt. SI	34.05	34.14
Rt. AP	17.29	17.43
Rt. ML	54.75	54.86
Rt. SI	33.89	33.95

*AP* antero-posterior, *ML* medio-lateral, *SI* superior-inferior

### Pre-operation displacement average

The result of the mean pre-operation displacement is shown in Table 2. The values in the superior-inferior direction were less than 2 mm. We therefore excluded the superior-inferior direction from analysis in our study, according to the direction of displacement, fixation methods, and post-reduction prognosis. We categorized the direction of displacement in the antero-posterior direction and medio-lateral direction.

**Table 2:** Pre-operation displacement

Measurement (mm)	Pre-operation displacement average	
	Mean	SD
AP	3.08	2.19
ML	2.96	2.40
SI	0.93	0.60

*AP* antero-posterior, *ML* medio-lateral, *SI* superior-inferior, *SD* standard deviation

### Classification according to the direction of displacement in pre-operation

We classified the direction of displacement as postero-medial, postero-lateral, antero-medial, and antero-lateral. The distribution (number and percentage) of cases in these categories according to the direction of displacement is shown in Table 3.

**Table 3:** Distribution according to the direction of displacement

Direction of displacement	Number (percentage)
Postero-medial	54 (53)
Postero-lateral	30 (29)
Antero-medial	14 (14)
Antero-lateral	4 (4)
Total	102 (100)

### Classification of the surgical approach

We also classified the surgical approaches used, as lateral canthotomy (we defined lateral canthotomy as all methods of fixation using a frontozygomatic suture), transconjunctival, lateral canthotomy + transconjunctival, Gillies method based on an intraoral approach, and an intraoral approach only. The distribution (number and percentage) of the various surgical approaches used is shown in Table 4.

**Table 4:** Distribution of the surgical approaches used

Surgical approach	Number (percentage)
Intraoral + lateral canthotomy	21 (21)
Intraoral + transconjunctival	5 (5)
Intraoral + lateral canthotomy + transconjunctival	24 (23)
Intraoral + Gillies	32 (31)
Intraoral	20 (20)
Total	102 (100)

### Crosstabulation analysis in the postero-medial direction post-operatively

In the postero-medial direction of displacement, the result of crosstabulation analysis of reduction was statistically significantly different among the surgical approaches. The result of crosstabulation analysis in the postero-medial direction is shown in Table 5.

**Table 5:** Result of crosstabulation analysis of the postero-medial direction post-operation

Reduction	Surgical approach					*χ* ^2^ (*P*)
	L	T	L + T	G	X	
Less than 2 mm	6 (50.0)	2 (100.0)	15 (93.8)	7 (43.8)	3 (37.5)	12.974* (.011)
More than 2 mm	6 (50.0)	0 (0.0)	1 (6.2)	9 (56.2)	5 (62.5)	
Total	12 (22.2)	2 (3.7)	16 (29.6)	16 (29.6)	8 (14.8)	

*L* intraoral + lateral canthotomy, *T* intraoral + transconjunctival, *L + T* intraoral + lateral canthotomy + transconjunctival, *G* intraoral + Gillies, *X* only intraoral

*Statistically significant difference between the groups (*P* < 0.05)

### Crosstabulation analysis in the postero-lateral direction post-operatively

In the postero-lateral direction of displacement, the result of crosstabulation analysis of reduction was statistically significantly different among the surgical approaches. These results are shown in Table 6.

**Table 6:** Result of crosstabulation analysis of the postero-lateral direction post-operation

Reduction	Surgical approach					*χ* ^2^ (*P*)
	L	T	L + T	G	X	
Less than 2 mm	1 (25.0)	2 (100.0)	5 (100.0)	3 (30.0)	4 (44.5)	9.711* (.046)
More than 2 mm	3 (75.0)	0 (0.0)	0 (0.0)	7 (70.0)	5 (55.5)	
Total	4 (13.3)	2 (6.7)	5 (16.7)	10 (33.3)	9 (30.0)	

*L* intraoral + lateral canthotomy, *T* intraoral + transconjunctival, *L + T* intraoral + lateral canthotomy + transconjunctival, *G* intraoral + Gillies, *X* only intraoral)

*Statistically significant difference between the groups (*P* < 0.05)

### Crosstabulation in the antero-medial direction post-operatively

In the antero-medial direction of displacement, the results of crosstabulation analysis of reduction did not differ significantly among the surgical approaches (Table 7).

**Table 7:** Result of crosstabulation analysis of the antero-medial direction post-operation

Reduction	Surgical approach				*χ* ^2^ (*P*)
	L	L + T	G	X	
Less than 2 mm	2 (40.0)	2 (100.0)	2 (40.0)	1 (50.0)	2.400 (.494)
More than 2 mm	3 (60.0)	0 (0.0)	3 (60.0)	1 (50.0)	
Total	5 (35.7)	2 (14.3)	5 (35.7)	2 (14.3)	

*L* intraoral + lateral canthotomy, *L + T* intraoral + lateral canthotomy + transconjunctival, *G* intraoral + Gillies, *X* only intraoral

*Statistically significant difference between the groups (*P* < 0.05)

### Crosstabulation analysis in the antero-lateral direction post-operatively

In the antero-lateral direction of displacement, crosstabulation analysis of reduction also did not reveal statistically significant differences among the surgical approaches, as shown in Table 8.

**Table 8:** Result of crosstabulation analysis of the antero-lateral direction post-operation

Reduction	Surgical approach				*χ* ^2^ (*P*)
	L	T	G	X	
Less than 2 mm	1 (100.0)	1 (100.0)	1 (100.0)	1 (100.0)	1.214 (0.673)
Total	1 (25.0)	1 (25.0)	1 (25.0)	1 (25.0)	

*L* intraoral + lateral canthotomy, *T* intraoral + transconjunctival, *G* intraoral + Gillies, *X* only intraoral

*Statistically significant difference between the groups (*P* < 0.05)

## Discussion

With ZMC fractures, displacement in the posterior direction was found to be the most frequent, while displacement in the superior-inferior direction was rare. These results coincide with those of Toriumi et al. [3] that indicated that displacement around the superior-inferior axis is the most frequent in ZMC fractures. In the natural condition, the zygoma arch breaks first when tripod fractures occur because it is the thinnest and the most fragile support of the zygoma. When this occurs, the zygoma remains supported at three sites: the frontal process, the inferior orbital rim, and the zygomaticomaxillary buttress. They hypothesized that fracture patterns are largely determined by which of these three remaining sites breaks first under trauma [3].

In this study, reduction results obtained using a transconjunctival approach were statistically superior to those obtained using an intraoral approach, a Gillies approach, or a lateral canthotomy approach for posterior displacement. Ellis et al. have shown that rotation of the entire complex along its vertical axis was noted, despite the presence of bone plates at the frontozygomatic and zygomaticomaxillary areas [1]. To prevent rotation of the entire complex, the sphenozygomatic area should be examined during surgery.

Karlan and Cassisi reported that the masseter muscle contributed significantly to the forces of mastication, which range from 11.25 to 90.00 kg. With ZMC fractures, when fixed at the zygomatic frontal suture, the fracture was found to rotate downward and backward with a masseteric force of less than 2.25 kg [11]. Hanemann et al. reported that the zygomaticofrontal suture seemed to be most affected by the action of the masseter [12].

Karlan and Cassisi showed that, geometrically, the three-point (frontozygomatic suture, infraorbital rim, and lateral maxillary buttress) alignment of zygoma fractures resulted in a more exact orientation of the zygomatic pyramid. Their abstract model as well as their analysis using moiré topographic maps of the skull demonstrated that downward, backward, and medial rotation of the fractured segment may still occur, despite a one-point or two-point alignment of the fractured segment [11].

Some surgeons have stated that a one-point fixation at the zygomaticomaxillary buttress is sufficient [13] for fixation in ZMC fractures. However, assessment of the orbital floor by using a transconjunctival approach is essential for repairing associated blowout fractures. Exposure of the lateral orbital wall also helps in identifying the alignment of the greater wing of the sphenoid bone [14].

Even when considering the limitations of the technique, Jo and Kim showed that treatment of zygomatic fractures by using a transconjunctival approach and an intraoral approach was advantageous, as it did not result in any incision scars and achieved favorable and stable anatomic and anthropometric outcomes. This approach could serve as a novel alternative for socially active young male or female patients who are sensitive to esthetic changes [14].

Davidson et al. have proposed that a two-point fixation using a miniplate alone conferred a degree of stability comparable to most three-point fixation methods, regardless of the site in which the miniplates were applied [15]. Based on this, Lee et al. asserted that a two-point miniplate fixation at the infraorbital rim and zygomaticofrontal suture would suffice in non-comminuted ZMC fractures [16].

The presence of diastasis in the zygomaticofrontal suture plays an important role when determining whether this portion should be exposed. Surgeons generally prefer to perform accurate reduction under full exposure, using a lateral brow incision. All patients in the present study had zygomaticofrontal suture displacement, although this displacement was not particularly severe [14].

We re-classified ZMC fracture displacement using the maximum projection coordinate of the zygoma as the classification criterion and assessed whether a specific approach for particular displacement of a ZMC fracture would enhance the esthetic results. However, we did not consider the severity of the ZMC fracture in this study. More complicated ZMC fractures require more approaches and fixations. A study considering fracture severity is therefore required for a more accurate analysis.

## Conclusions

After ZMC fracture, displacement in the posterior direction was the most frequent; however, displacement in the superior-inferior direction was rare. Reduction using a transconjunctival approach and an intraoral approach simultaneously produced statistically better results than using an intraoral approach, Gillies approach, or lateral canthotomy approach for posterior displacement. Further similar studies are required for anterior displacement after ZMC fracture. However, when posterior displacement occurs, using an intraoral approach and transconjunctival approach simultaneously is recommended for reduction and accurate fixation of the displaced fragment.
